# The Neuroendocrine System and Stress, Emotions, Thoughts and Feelings**

**DOI:** 10.4103/0973-1229.77430

**Published:** 2011

**Authors:** George E. Vaillant

**Affiliations:** * *Harvard Medical School, 1249 Boylston St, Boston, MA 02215, USA.*; ** *Revised and peer reviewed version of a Paper read at an International Seminar on Mind, Brain, and Consciousness, Thane College Campus, Thane, India, January 13-15, 2010.*

**Keywords:** *Positive emotions*, *Limbic system*, *Spiritual*, *“Right-brain”*

## Abstract

The philosophy of mind is intimately connected with the philosophy of action. Therefore, concepts like free will, motivation, emotions (especially positive emotions), and also the ethical issues related to these concepts are of abiding interest. However, the concepts of consciousness and free will are usually discussed solely in linguistic, ideational and cognitive (i.e. “left brain”) terms. Admittedly, consciousness requires language and the left-brain, but the aphasic right brain is equally conscious; however, what it “hears” are more likely to be music and emotions. Joy can be as conscious as the conscious motivation produced by the left-brain reading a sign that says, “Danger mines!!” However, look in the index of a Western textbook of psychology, psychiatry or philosophy for positive emotions located in the limbic system. Notice how discussion of positive spiritual/emotional issues in consciousness and motivation are scrupulously ignored. For example, the popular notions of “love” being either Eros (raw, amoral instinct) or agape (noble, non-specific valuing of all other people) miss the motivational forest for the trees. Neither Eros (hypothalamic) nor agape (cortical) has a fraction of the power to relieve stress as attachment (limbic love), yet until the 1950s attachment was neither appreciated nor discussed by academic minds. This paper will point out that the prosocial, “spiritual” positive emotions like hope, faith, forgiveness, joy, compassion and gratitude are extremely important in the relief of stress and in regulation of the neuroendocrine system, protecting us against stress. The experimental work reviewed by Antonio Damasio and Barbara Fredrickson, and the clinical example of Alcoholics Anonymous, will be used to illustrate these points.

## Introduction

This paper will suggest that the prosocial, “spiritual” positive emotions like hope, faith, forgiveness, joy, compassion and gratitude are extremely important in the relief of stress and in regulation of the neuroendocrine system. The positive emotions protect us against stress. In doing so, I wish to emphasise that there is more to the mind than the scientific and the philosophical. There is the “spiritual.” The Greek citizens of Corinth knew about philosophy from Plato, science from Archimedes and negative emotions from their rather unspiritual religion. However, to learn about faith, hope and love, they had to wait 300 years for a Jewish Pharisee, later named St. Paul. To understand the mind, the Hindus, adept at both science and philosophy, had to wait for the Bhagavad-Gita.

On the other hand, if we wish to understand the human organism, consciousness may be the wrong place to start. Consciousness is, perhaps, an illusion of the left-brain language centres. I think (in words) therefore I am. That is to risk being wrong about many things. Like the fire’s visible flame, thought is merely a phantom of the nonverbal passion/combustion going on within the limbic system beneath the verbal cortex. The moral is do not believe everything you think.

Brain scientists dismiss philosophers for indulging in speculation devoid of empirical evidence and hence unjustly accuse them of ‘talking thru their hat.’ And so, the philosophers rightly worry that brain research, such as will appear in my paper, will only touch the fringe of what goes on in the mind and consciousness (Singh and Singh, 2011; p36).

Philosophy of mind is intimately connected with the philosophy of action. Therefore, concepts like free will, motivation, emotions (especially positive emotions), and also the ethical issues related to these concepts are of abiding interest (Singh and Singh, 2011; p10). However, the concepts of consciousness and free will are usually discussed solely in linguistic, ideational and cognitive (i.e., “left brain”) terms. Admittedly, consciousness requires language and the left brain, but the aphasic right brain is equally awake; however, what it “hears” are more likely to be music and emotions difficult for our philosophy to encompass.

Admittedly, sometimes right-brain activities like joy and music can be just as conscious as the conscious motivation produced by the left-brain reading a sign that says, “Danger mines!!” But too often, action tendencies, like defence mechanisms or the results of meditation or transference and tantrums, can be unconscious, but very real. Russell D’Souza characterised the dichotomy between the spiritual and scientific mind, “The Buddha’s enlightenment was considered too good to be true, but now Western enlightenment is being seen as too true to be good.” (D’souza and George, 2006). Sociobiologist, Edward O. Wilson (1998) puts it a little differently: “The essence of humanity’s spiritual dilemma is that we evolved genetically to accept one truth and discovered another” (p264). With the printing press and then the Enlightenment, positive emotion became subordinated to lexical neocortical science and dogma. However, as the French discovered with their atheistic guillotine, the Enlightenment was too true to be good.

## Evolutionary Basis For The Positive Emotions

Put differently, neocortical science and limbic spirituality both mediate survival, but to do so they depend on different parts of our highly integrated brain. One truth, however, cannot be truer than another.

Let me begin with the example of attachment. The last 200 million years of the evolution of the human mind would have been inconceivable without the concept of attachment. However, until the 1950s, attachment was invisible to philosopher and scientist alike. Autism, a congenital defect of attachment was not even discovered until 1943. It was not recognised in our diagnostic nomenclature until the 1990s. Freud spoke of Eros, neuroscientists talked about hypothalamic motivated sexual behaviour and theologians spoke of Agape. But Love (i.e., attachment) is selective and enduring. Eros is not enduring and agape is not selective. Both the scientists and the philosophers were talking through their hat. It took two ethologists (masters of a brand new science), Harry Harlow and John Bowlby, to put attachment on the map (Harlow, 1958; Bowlby, 1979).

The popular notions of “love” being either Eros (raw, amoral instinct) or agape (noble, nonspecific valuing of all other people) miss the motivational forest for the trees. Unlike Eros, attachment spells Love, even when your spouse has “a headache.” Attachment has nothing to do with consciousness and everything to do with community; and community, as the “East” knows and the “West” forgets, is at the basis of brain evolution. I was raised in an elite American boarding school on Kipling’s mantra, “He travels fastest who travels alone.” But when I became a grandfather, and turned my laboratory over to a young colleague and retreated to the beaches of Australia to seek the meaning of the mind, I discovered a new mantra from a man, who like Kipling, had cut his literary teeth in India but who had the wit to listen. As they say in AA, E.M. Forster took the cotton out of his ears and put in his mouth.

My new mantra was “Only connect. Only connect the prose and the passion and both will be exalted and love will be seen at its height” (Forster, 1910).

Even in the 21^st^ century, look in the index of a Western textbook of psychology, psychiatry or philosophy for positive emotions. Notice how discussion of positive spiritual/emotional issues in motivation is scrupulously ignored. Ignored is the Saint-Exupery’s little fox warning: Only the heart sees rightly; what is important is invisible to the eye (Saint Exupery, 1943).

Attachment is deeply motivational but is absent to a remarkable degree in brilliant autistics and in the writings of the brilliant Sigmund Freud. And yet, attachment and social emotional intelligence are present in low IQ children with Down’s syndrome. Attachment has nothing to do with intelligence and everything to do with community.

Let me offer a list of eight positive emotions: love, awe, hope, compassion, faith (trust), forgiveness, joy and gratitude to suggest that they are the building blocks of human attachment, community and spirituality.

A leitmotif throughout this paper will be to wonder why religions, in contrast to scientists and philosophers, find it so much easier to pull the positive emotions up into consciousness. One might even say that Sartre, Wittgenstein, Freud and Skinner did not do positive emotions. In 1956 at Harvard Medical School, the only emotions I learned about were the hypothalamic negative emotions: fight, flight, hunger and lust. All were rooted in time present and all were about me.

It is attachment, a mammalian emotion that, even more than consciousness and “free will,” is future oriented, not about the self, and plays horse to the cart of conscious thought in drawing action forward. It is the limbic positive emotions, not the hypothalamic negative emotions, which distinguish human beings from reptiles. In contrast to negative emotions, positive emotions are all about the other and all about the future, and that is why the mind evolved, so that it could build communities.

So why, in greater detail, did natural selection create positive emotions? Mammalian evolution had begun in the dark to protect little furry insectivores from hungry, sun-loving carnivorous reptiles. At first, their sense of smell was as or more important than sight. Thus, these nocturnal mammals possessed a highly developed limbic olfactory system (the rhinencephalon) or smell brain. In order to find food and to remain connected to each other in the dark, a good sense of smell was a necessity. Some scientists (Panksepp, 1999) have called this smell brain (a.k.a. limbic system) the “seeking system.” The seeking, however, is about connection to one’s fellow insectivores as much as to food or sexual discharge per se. With equal importance, the brains of mammals, relative to their body size, began to grow. Unlike the case for dinosaurs and fish, increasing brain complexity, instead of traits like size, teeth and bright colours, was selected for in mammals.

With time, some of these early mammals evolved into light-loving creatures for which a stereoscopic visual system and improved hearing reshaped the responsiveness of their smell brain. Primates, and many other mammals, now use their former smell brain to stay in touch with their mates vocally and visually rather than by odour, but the limbic language of attachment still defies translation into English or Urdu. Instead, human beings become quite inarticulate when they try to describe what they smell or whom and why they love. Attachment depends upon body language, scents, vocal timbre and lullabies, not the language of the neocortex. We confabulate when we try to put the scent of an orchid, the nose of a great burgundy wine or a life-altering spiritual experience into words.

Indeed, language, like too articulate religions, often separates human beings. In contrast, emotions, body language, facial recognition, touch, pheromones and the spirituality of a limbic smell brain often bind us together. Through discriminating audition, the kitten’s mew or the human infant’s separation cry evokes unselfish love in almost all of us. Thus, from the limbic system and the temporal neocortex that it serves comes the very sort of information provided in hymns, psalms and love letters–emotional, musical, mystically important information. Such information is very different from that contained in almanacs, science journals and philosophical treatises.

For example, the limbic separation cry, mediated unconsciously by the anterior cingulate gyrus, advertises vulnerability and distinguishes mammals from fish and reptiles. Mammalian evolution has led to an intricate three-boned (malleus, incus, stapes) apparatus in the inner ear that permits rodent mothers to hear their infants’ high-pitched cries inaudible to predator birds and reptiles. The separation cry presupposes a hard-wired emotional trust in a maternal protector who will find you, feed you and protect you–a maternal guardian who unlike a father reptile or mother fish will not just find you and gobble you up.

The nature of human unselfish love becomes still clearer if we reflect upon life in the African savannas one or two million years ago. On those sparsely wooded plains evolved our hairless ancestors who took several years to reach maturity. Although they lived in a land richly endowed with carnivores, our ancestors could not run like the gazelle, burrow like the rabbit, climb trees like the gibbon, fly away like the flamingo or fight back like the elephant. If human beings did not band together, they perished. Human beings do not even have fur like the ape for the young to cling to; instead, the human mother must cling to her young. In order to survive, human beings had sometimes to subordinate both hunger and sex per se to the development of an inborn altruistic social organisation. From such social bonding came lasting attachment and the survival of their young. On the savannah, a young gazelle can survive with “selfish genes.” As soon as it is born, it can walk. In contrast, if not born into an unselfish human community, the Homo sapiens child is destined to become some predator’s lunch.

The increased brain size and prolonged dependency of evolving human beings each catalysed the other. Unconditional and forgiving love became essential to human survival, but such attachment is not rigid and reflexive as is the gosling’s imprinting on its mother goose or as is a mother bluebird’s stereotyped care of her young. Rather, love in primates depends on emotionally motivated decision making and on flexibility, if not quite “free” choice. Thus, unlike geese and blue birds, human beings have developed religious memes to reinforce their care-taking behaviours. But all this required an increasingly complex and, thus, a larger brain. And mirror cells in the insula, buried away from the notice of even twentieth century neuroscience and the loveless philosophies of Sartre and Hobbes, made us all empathic beings, however buried in the unconscious.

## Mirror Neurons and Spindle Cells

While witnessing a loved one’s pain, our own limbic emotional centres for pain are aroused, but not our neocortical analytical centres that would effect motor avoidance were the pain our own. Putting it differently, when witnessing another person burning their hand, the “mirror” neurons in our own limbic insula and anterior cingulate “light up” on the neuroimagist’s screen as if the hand was our own. But the cells in our neocortical analytic and motor centres (e.g., “I feel a burning in my left hand that prompts me to pull it away”) remain quiescent (Singer et al., 2004). Of interest is that such neurological brain activation when witnessing another’s pain correlates significantly with the observer’s scores on pencil and paper tests assessing empathy.

The higher apes are also set apart from other mammals by a unique and newly evolved neural component called the spindle cell. Human beings have twenty times more spindle cells than either chimps or gorillas. (Adult apes average about 7,000 spindle cells, human newborns have four times as many and human adults almost thirty times more spindle cells.) Monkeys and other mammals are without these special cells. These large cigar shaped “spindle” or “Von Economo” neurons appear central to the governance of social emotions and moral judgment (Allman et al., 2001). These cells may help us to feel human connection and indirectly to reflect upon and act on that feeling. Spindle cells may have helped the great apes and human beings integrate their mammalian limbic system with their expanding neocortices.

In recent imaging tests, spindle cells have been shown to light up in our skulls like summer evening fireflies in response to a variety of different emotional and social stimuli: the picture of a loved one; scenes of others suffering; feelings of personal embarrassment, or guilt or self-consciousness.

And yet, as integral as these specialised neuronal cells seem to be to our very identity as human beings, they are not even present in our brains at birth. They only begin to emerge at about the fourth month of life and, over the course of the next four years or so, continue to grow and migrate toward their permanent home in the right frontal cortex, weaving themselves into place there in direct concert with our newly emerging sense of self–our feelings of devotion, compassion and remorse; our sense of right and wrong: the early fabric, in effect, from which we go on to weave our own individuality and personal life story. Spindle cells exist in the anterior cingulate cortex, the prefrontal cortex and the insula, a still somewhat mysterious region of the limbic system that may facilitate empathy. In brain imaging studies, the insula lights up “when people look at romantic partners, perceive unfairness … experience embarrassment, or if they are mothers, hear infants cry” (Blakeslee, 2003). In short, the limbic anterior cingulate and insula appear active in the positive emotions of humour, trust and empathy. Neurologically, the insula is also closely bound to bringing into consciousness sensations in the heart - literally and metaphorically.

## Social Bonding

In short, human beings have survived by sophisticated social bonding–characterised by unconditional attachment, forgiveness, gratitude and affectionate eye contact. And both the philosophers and scientists ignore them. Often, eye contact is more important than speech in guiding behaviour but entirely unconscious. As the popular song goes “Your lips tell me, “No. No!” but there is “Yes, Yes!!” in your eyes.” I doubt that Gandhi’s passive resistance would have worked with Hitler and Stalin. How did he consciously know that he had read the “eyes” of verbally adamant Churchill’s imperialism right? In our deliberations here, we need to let our musical right brains in on our conversation.

An interesting bodily reflection of human beings’ shared intentionality is the sclera, or whites, of the eyes. All 200 or so species of primates have dark eyes and a barely visible sclera. All, that is, except human beings, whose sclera is three times as large, a feature that makes it much easier to follow the direction of someone else’s gaze. Chimps will follow a person’s gaze, but by looking at his head, even if his eyes are closed. Babies follow a person’s eyes, even if the experimenter keeps his head still.

True, the negative, but self-consuming, emotions of disgust, anger, fear and envy have often allowed individual human beings to push our enemies away or to selfishly exploit them. The positive emotions, however, of love, joy, hope, forgiveness, compassion and trust have allowed human beings to draw close to one another and to survive more successfully. Yes, fear draws people together too–but without the sharing.

Evolution has liberated human love from the reflexive neuroendocrine dominance by the hypothalamus and instead has made mate choice and bonding based on relatively flexible motivation. The “moral,” mature, and cause and effect mentalisation within the frontal lobes takes over from adolescent hypothalamic impulse.

With maturity, empathic attachment replaces sexual greed. Thus, contrary to current Western fashion, not only have arranged marriages been far more common in human history than “marrying for love,” but follow-up of such arranged marriages reveals them to be as enduring. The elderly widow from a Hindu arranged marriage grieves as deeply at her husband’s funeral pyre as does any modern married-for-love London widow at a suburban funeral home. Human attachment takes time.

## How Do We Learn Love?

So, how do we learn love? How do we become agents of love? Not through Sunday school, not through the Internet, not ever by words alone. We learn to love through neurochemistry, genes and identification.

In part, enduring selective love is catalysed by genes. The crippling social limitation of infantile autism is almost wholly genetic. For unknown, but highly heritable reasons, autistic individuals are unable to take love in and thus unable to give love back. However, enduring love in human beings is different from that in insects. Insects have genetic communication systems for “altruistic” behaviours that are sometimes impressively sophisticated, but they neither invent them nor teach them to others. The waggle dance of the honeybee and the odour trail of ants contain symbolic elements, but their altruistic performance and meaning are genetically inborn and cannot be altered by learning. Unlike human compassion, the genetically mediated “altruism” of insects is not culturally contagious.

In part, enduring selective love is catalysed by chemistry. Neurochemistry provides ingenious models of nonverbal communication and catalyses the involuntary mechanisms of positive emotion. The brain hormone, oxytocin, is released when all mammals give birth. Oxytocin seems to permit mammals to overcome their natural aversion to extreme proximity, and thus, oxytocin has been popularly rechristened the “cuddle hormone.” If they are genetically deprived of oxytocin, monogamous, maternal, loving prairie voles (a species of rodent) turn into another subspecies–the heartless, promiscuous, pup-abusing montane voles. Without oxytocin, parental cooperation and responsibility vanishes (Shapiro and Insel, 1990; Insel, 2002). In human newborns, there is a short-lived overproduction of oxytocin receptors (Davidson and Harrington 2002, p. 116). Oxytocin goes up in human puberty in parallel with adolescent crushes. Put a newborn baby in a mother’s arms or bless a couple’s sexual union with mutual orgasm and brain oxytocin levels rise.

The oxytocin-rich dopaminergic brain centres are an intimate part of the human limbic system. The nucleus accumbens (in voles), the ventral tegmentum (in rats), the anterior cingulate gyrus (in human beings) have all been shown to be closely involved in lasting mammalian attachment. All are heavily dependent on the neurotransmitter dopamine; and interestingly, these same three brain centres also contain opiate receptors and are linked to heroin addiction-an ersatz and often lethal “love”–that is also selective and enduring. Opiates are the only chemicals that can comfort a baby animal separated from its mother. Or as one chronic addict described it, “You don’t really get lonely on smack [heroin]. It’s like having a lover” (Edwards, 2004, p. 138).

In part, enduring selective love is catalysed by identification. Unlike honeybees, human beings do not come into the world knowing how to dance. If all human love is a dance, it still takes two to tango–and usually at the beginning an experienced tango teacher. Thus, chemistry, genes and survival of the fittest are only part of the story. True, the evolutionary march from fish to cold-blooded reptiles to Harry Harlow’s loving monkeys reflects the power of genes to lay the groundwork for love. However, for enduring mammalian attachment to occur loving environments and identification with others are as critical as chemistry to sculpt the brain.

If as the French planter sings in South Pacific “you have to be taught to hate and fear,” you also have to be shown how to love. Thus, the behavioural self-regulation that we associate with love does not come from a solitary brain, but from one’s brain evolving and becoming shaped through attachment to a beloved other. Monkeys raised in isolation go on eating binges and cower in corners. Instead of playful roughhousing, they fight with their peers unto death, and they never really get the hang of copulation. All their lives, such isolated monkeys remain inept “at doing what comes naturally.” In contrast, isolated monkeys who are subsequently raised by mothers or with siblings for even one year can learn to roughhouse–gracefully stopping once social dominance is achieved–and skilfully negotiating the dance steps necessary for successful impregnation (Harlow, 1958).

As the parables, hymns and uplifting narratives of the world’s great religions suggest that the biology of love is catalysed by social example. This was demonstrated in a series of ingenious experiments by University of Virginia psychologist, Jonathan Haidt and his students. By showing new mothers video clips reflecting love and gratitude, they increased the leakage of milk and/or nursing behaviour (both evidence of oxytocin release). These effects were much less evident if the mothers were shown humorous or neutral videos. Again, when they showed college students documentaries of heroic altruists and uplifting video segments illustrating displays of gratitude and unselfish love, Haidt and colleagues evoked in the students a sense of calm, a warm feeling in the chest, and an impulse to help others, not in evidence after the same students viewed neutral video clips (Haidt, 2006).

Love, especially unconditional love, also cures people–both those who give it and those who receive it. Love, like the other positive emotions, is religion without the side effects. Healing love, of course, always involves appropriate boundaries. Eye contact and touch, as in mother-child interaction, must always be kept separate from lust and selfish Eros, or the other person will feel violated. The good hospice nurse, the committed parish priest, the dedicated caseworker, even a best friend needs to remember that a favourite grandmother, not a charismatic lifeguard, is the proper model for the connectedness, the passion, the commitment and the wise limits that create therapeutic love. Healing love is often more about witnessing (making the other person feel “seen”) than about rescuing (Herman, 1997).

Moreover, oxytocin, the “cuddle hormone,” in some ways is itself as remarkably healing as the love that it underlays. Over the long-term, oxytocin exerts effects opposite to the negative “fight-flight” emotions. During prolonged periods of fear, anxiety and depression, pain thresholds are lowered and cortisol levels and blood pressure can be chronically and deleteriously elevated. In contrast, during periods of sustained oxytocin release, cortisol levels and blood pressure are reduced, pain thresholds are increased and a calm nonanxious state results (Uvnas Moberg, 2003). A recent study of the cortisol elevation and post-stress anxiety involved in public speaking found that intranasally administered oxytocin and social supports, each buffered the effects of stress, and they were most effective when given in combination (Heinrichs *et al*., 2003). No wonder love and compassion are valuable at the bedside of the sick.

Let me illustrate this by suggesting that the unconscious heart sees more meaningfully than the conscious eye. The sympathetic nervous system is appealing to the Western mind. Fight, Compete, all blood to the muscles and away from the gut, glucose stores mobilised, Yay! But we are also talking about the roots of the metabolic syndrome (hypertension, hypercortisolmia and secondary diabetes).

Look at the parasympathetic nervous system. Nothing, but boring Buddhist, “rest and digest.” Yes, meditation slows the heart and basal metabolic rate better than sleep. But the parasympathetic nervous system is also at the root of Barbara Fredrickson’s landmark “broaden and build” research. By soothing research subjects with videos of positive emotions (you can do it at home just by cuddling), she could lower pulse rate, speed cardiac recovery and enhance memory, creativity and social tolerance. Stimulate the sympathetic nervous system with Hobbesian negative emotion and you reverse the process.

Spirituality is the amalgam of the positive emotions that bind us to other human beings–and to our experience of “God” as we may understand Her/Him (Vaillant, 2008). Love, hope, joy, forgiveness, compassion, faith, awe (Keltner and Haidt, 2003) and gratitude (McCullough et al., 2001; Emmons, 2007) are the eight spiritually important positive emotions addressed here.

I have omitted from the list four other positive emotions–excitement, contentment, mirth and a sense of mastery, as we can feel these latter four emotions alone on a desert island. In sharp contrast, the eight positive emotions that I have selected all involve human connection. None of the eight are all about “me.” All stimulate the parasympathetic nervous system.

Let me carry this hypothesis further. Dopaminergic brain tracts can be shown to underlie addictive behaviour in mammals and reptiles. A scientist can produce pleasure in the brain by inserting dopamine into the primitive brain circuitry that links the “reptile” brain’s addiction centre, the nucleus accumbens and the superior tegmentum.

But in mammals, these same dopaminergic tracts run from the mid-brain–the reptile brain–to the limbic system–the part of the mammalian brain that serves attachment. Then, the transmission continues to the anterior cingulate gyrus-that part of the mammalian limbic cortex that makes the past emotionally meaningful. In mature human beings, the same dopaminergic tracts travel to the most recently evolved portions of the orbitomedial frontal lobes that serve planning, empathy, morality and a mother’s smile as she gazes at her baby. Or in the words of Thomas Insel, NIMH Director, “It is also possible that neural mechanisms that we associate with drug abuse and addiction might have evolved for social recognition, reward and euphoria–critical elements in the process of attachment” (Insel and Young, 2002).

In the words of a sensitive observer, social psychologist, Mihaly Csikszentmihalyi, (Csikszentmihalyi, 1990, p. 21), religious “practices get overgrown by brambles of meaningless mumbo jumbo. Ritual form wins over substance and the seeker is back where he started.” In contrast, mature faith always understands that all spirituality is a journey, and thus, life remains inherently developmental. One thing becomes another; one never arrives. Developmentally, organised religion provides faith’s portal; deep spirituality remains the prize. Like good science, mature faith can distinguish the forest from the trees. As Gandhi commented to an English friend “I don’t think much of your Christianity but I like your Christ” (Singh and Singh, 2004).

Let me close with a cheer for consciousness and language, but only when linked with the limbic positive emotions. As a corollary of cortical brain expansion in human beings, came an increasingly focused consciousness. True, an eagle’s sharp eye can instinctively discriminate a distant stone from a distant mouse better than a human eye. But human beings can reflect upon the distinction and can bring the question of feelings (Am I hungry?) up into reflective consciousness. Armchair critics can scoff at the follies of modern judicial punishments, but for the last 30 centuries through the conscious reflection upon the long-term consequences of angry retaliation, the deterrence of criminal behaviour has become progressively more rational and more loving. We are still learning! We may moan about our urban murder rate, especially in the American and Brazilian cities with all their handguns. But in fact, since the 13th century, the murder rate has gone steadily down until it is only 2% of what it was 700 years ago (Levitt and Dubner, 2006).

The evidence for convincing positive cultural evolution of Homo sapiens sapiens is far more convincing. About 30,000 B.C. the last, physically more powerful and bigger brained Neanderthals expired–not with a bang but a whimper–in remote caves at the tip of Spain. In the place of robust strength, came the evolution of successful social organisation that depended upon capacity to plan for the future, shared awe through artistic creativity and an increasing concern for the sick and the lame.

The critical difference was the genetic mutation about 170,000 years ago that gave the Neanderthal’s African (cro-magnon) cousins, from whom we are all descended, the gift of language and thus the capacity for cultural evolution.

Over the past 20,000 years, this inexorable march of spiritual development, artistic skill and culturally mandated unselfish care of the weak has continued to evolve. Evidence of organised religion accompanied evidence of stable settlements seven to twelve millennia ago. However, until the transformative millennium, a millennium extending from 600 B.C. to 700 A.D., the world’s great cities emerged only to disappear. Ur, Babylon, Mohenjo-Daro, Carthage, Thebes, Machu Picchu, the Mayan metropolis of Tikal and the early Chinese capitals vanished beneath sand, fields and jungle creepers. Not until the Bhagavad-Gita, Buddhism and Christianity became established, not until organised religions that emphasised love and compassion rather than fear and dominance, did great cities endure. The early Benedictine monks followed the principle that the care of the sick is to be placed above and before every other duty.

Richard Dawkins and Adolf Hitler would call that a really dumb way to pass on one’s gene pool. Thus, the selfish but very “fit” and scientifically advanced Third Reich took a dim view of the sick and believed that a society’s resources should be devoted only to the genetically healthy and to selfish conquest. In order to decide whether the Nazi or Benedictine faith is better suited to a Darwinian perspective, we must depend not upon soft-hearted “liberals” battling the sharp wits of the right, but we must depend upon science–upon empirical long-term follow-up. The Nazi order lasted barely a decade, but after 1500 years, the Benedictine Order is still alive and well. The brilliantly rational but spiritually challenged French Revolution lasted no longer than the Third Reich. In short, I would conclude that positive emotions of the loving irrational limbic system are just as important to cultural survival as is the ingenious and rational neocortex.

The evolution of that very Franciscan “instrument of peace” –the universally prestigious Nobel Peace Prize–has taken place only in the last century. In dramatic contrast, over the last two million years, the genetic evolution of the human hypothalamus with its capacity for the 4 Fs–fight, flight, feeding and fornication“is only modestly more sophisticated than an alligator’s. Human capacity for time present negative emotions like fear, disgust and attack has probably not evolved much beyond that of a cornered rat. However, our capacity for the future-oriented positive emotions like joy, the Samaritan response, hope and forgiveness continues to evolve. Human beings, for better or for worse, remain a work in progress. It took patience and long-term follow-up, but Rudyard Kipling studying today’s financial news would be astonished to find the Indian economy buttressed by millennia of deep spirituality was faring better than the Hobbesian British financial establishment.

Another clear example of the survival value of spirituality and positive emotions in the modern world was the decade of 1975 to 1985 in Cambodia. In 1975, the Khmer Rouge gained absolute control of the country, and systematically, and for most idealistic “Marxist” reasons in the world, tried to abolish Buddhism and familial love. Sentimental attachment to a family member or to a temple was believed to impede rational rapid social progress and was punishable by death. Pol Pot’s idealistic regime hoped to instil in young children, separated from their families, an attachment to agrarian simplicity and to create a society without memory of urban decadence, monastic indolence or even money. Thoreau, Jefferson and Gandhi might have possibly admired Pol Pot’s ends–but not his means.

Four years later when the Khmer Rouge regime fell, the Cambodian children, now orphans, remained passionately attached to what remained of their extended families; and Buddhism rapidly asserted itself as a high point of village life. It was not from do-gooder central planning, it is how the human brain has evolved to work.

## Concluding Remarks [see also Figure 1]

**Figure 1:**
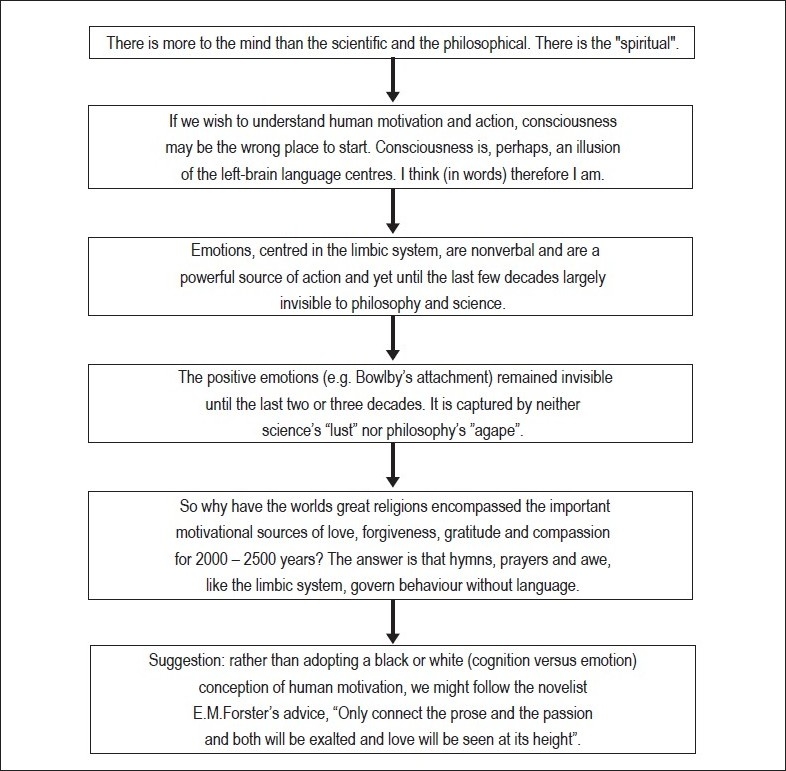
Flowchart of paper

The evolution of human beings has been much more moral and spiritual than the philosopher Hobbes and the geneticist Richard Dawkins give it credit for. The philosophical discovery of the positive emotions, of course, is 2500 years old. The Gita, the Pali Canon and the Old Testament spell out the importance of love, compassion, forgiveness and gratitude. But only in the last 20 years have the social and biological sciences assembled the necessary techniques to convince the cognitive mind of what the heart has always known (Vaillant, 2008).

### Take home message

Happiness is love. Period! (The fifty-year prospective follow-up empirical evidence for this extraordinary generalisation is in press)

## Questions That This Paper Raises

1. Do we need to use brain imaging to clarify more fully the differences between attachment to drugs and attachment to people?
2. Can we use brain imaging to further delineate differences between positive emotions like joy, hope, trust and gratitude?
3. What is the significance of Fredrickson’s work on linking the autonomic nervous system to different positive emotions, and do we need to further it?

## About the Author

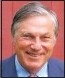



George E. Vaillant is a Professor of Psychiatry at Harvard Medical School and the Department of Psychiatry, Brigham and Womens Hospital. Dr. Vaillant has spent his research career charting adult development and the recovery process of schizophrenia, heroin addiction, alcoholism and personality disorder. He has spent the last 35 years as Director of the Study of Adult Development at the Harvard University Health Service. The study has prospectively charted the lives of 824 men and women for over 60 years. His published works include Adaptation to Life, 1977; The Wisdom of the Ego, 1993 and The Natural History of Alcoholism - Revisited, 1995. His summary of the lives of men and women from adolescence to age 80, called Aging Well, was published by Little, Brown in 2002. His latest book Spiritual Evolution: A Scientific Defense of Faith is in press. A graduate of Harvard College and Harvard Medical School, Dr. Vaillant did his residency at the Massachusetts Mental Health Center and completed his psychoanalytic training at the Boston Psychoanalytic Institute. He has been a Fellow at the Center for the Advanced Study in the Behavioral Sciences and is a Fellow of the American College of Psychiatrists. He has been an invited speaker and consultant for seminars and workshops throughout the world. A major focus of his work in the past has been on developing ways of studying defence mechanisms empirically. More recently, he has been interested in successful aging, positive emotions and spirituality. He is on the steering committee of Positive Psychology. He is also on the Honorary International Editorial Advisory Board of MSM. Dr. Vaillant has received the Foundations Fund Prize for Research in Psychiatry from the American Psychiatric Association, the Strecker Award from the Institute of Pennsylvania Hospital, the Burlingame Award from The Institute for Living and the Jellinek Award for research in alcoholism. He has twice received research prizes from the International Psychogeriatric Society. Most recently, he received the distinguished service award from the American Psychiatric Association.
